# Quantification of microbial degradation activities in biological activated carbon filters by reverse stable isotope labelling

**DOI:** 10.1186/s13568-019-0827-0

**Published:** 2019-07-16

**Authors:** Xiyang Dong, Leonard E. Bäcker, Mona Rahmatullah, Daniel Schunk, Guido Lens, Rainer U. Meckenstock

**Affiliations:** 1 Biofilm Centre, University of Duisburg-Essen, Universitätsstrasse 5, 4514 Essen, Germany; 2 RWW Rheinisch-Westfälische Wasserwerksgesellschaft mbH, Am Schloß Broich 1-3, 45479 Mülheim, Germany; 3 Present Address: School of Marine Sciences, Sun Yat-Sen University, Zhuhai, 519000 China

**Keywords:** Biological active carbon filters, Drinking water production, Dissolved organic carbon, Microbial degradation, Stable carbon isotopes

## Abstract

Biological activated carbon (BAC) filters are frequently used in drinking water production for removing dissolved organic carbon (DOC) via adsorption of organic compounds and microbial degradation. However, proper methods are still missing to distinguish the two processes. Here, we introduce reverse stable isotope labelling (RIL) for assessing microbial activity in BAC filters. We incubated BAC samples from three different BAC filters (two granular activated carbon- and one extruded activated carbon-based) in a buffer amended with ^13^C-labelled bicarbonate. By monitoring the release of ^12^C–CO_2_ from the mineralization of DOC, we could demonstrate the successful application of RIL in analysing microbial DOC degradation during drinking water treatment. Changing the water flow rates through BAC filters did not alter the microbial activities, even though apparent DOC removal efficiencies changed accordingly. Microbial DOC degradation activities quickly recovered from backwashing which was applied for removing particulate impurities and preventing clogging. The size distributions of activated carbon particles led to vertical stratification of microbial activities along the filter beds. Our results demonstrate that reverse isotope labelling is well suited to measure microbial DOC degradation on activated carbon particles, which provides a basis for improving operation and design of BAC filters.

## Introduction

Dissolved organic carbon (DOC) is composed of complex mixtures of heterogeneous organic compounds derived from the decay of organic matter and other sources. In drinking water production from surface water such as rivers and barrage lakes, it is important to remove the DOC because it affects the drinking water quality and performance of water treatment processes (Korotta-Gamage and Sathasivan 2017a, b; Li et al. 2017). Part of the DOC is biodegradable dissolved organic carbon (BDOC) that can serve as substrate for bacterial growth in the distribution network and may be responsible for undesirable color, taste or odor of drinking water. A widely used technology for DOC removal in drinking water treatment plants is ozonation followed by activated carbon filtration (Hong et al. 2018; Korotta-Gamage and Sathasivan 2017a). The refractory fraction of DOC can be partially oxidized in the ozonation step, which greatly improves the biodegradability (von Gunten 2003). The next step is a passage through an activated carbon filter which serves both as adsorbent for the DOC and as a support for microbial growth. Thus, microorganisms can populate the activated carbon filter where they degrade the natural BDOC, but also the BDOC produced by ozonation of the total DOC.

Hence, the activated carbon (AC) filters become biological activated carbon (BAC) filters, possessing both the adsorption capacity of the AC and the biodegradation potentials of the microorganisms colonizing the surfaces and macropores of AC particles (Aryal and Sathasivan 2011; Korotta-Gamage and Sathasivan 2017a). A major benefit of the microbial degradation is the significantly prolonged lifetime of the activated carbon filters before they need to be regenerated (Gibert et al. 2013; van der Aa et al. 2011). The degradation rate of adsorbed organic carbon thus becomes an important parameter in the purification process, which is, however, mostly not measured in drinking water production plants. This is partly due to the lack of suitable and reliable methods to assess biodegradation processes on solid matrices. Nevertheless, culture-independent molecular tools have shown that highly diverse microbial communities of bacteria and archaea populate BAC filters (Gulay et al. 2016; Vignola et al. 2018).

Most of the recent studies could only indirectly describe microbial activities on AC filters by measuring e.g. biomass densities (Fonseca et al. 2001), adenosine tri-phosphate (ATP) content (Gibert et al. 2013; Velten et al. 2007), or quantifying microbial cells with epifluorescence microscopy (EFM) or flow-cytometry (Vignola et al. 2018). So far, only a few laborious and cost-ineffective methods can directly determine the microbial activity of biofilms (i.e. organic compound degradation rates) on activated carbon particles, e.g. by monitoring bacterial uptake of labelled substrates or consumption of dissolved oxygen using a respirometric method (Servais et al. 1991; Urfer and Huck 2001). Very sensitive options to assess biodegradation are stable isotope-based methods, which employ either fully or partially labelled substrates such as ^13^C-glucose (Saito et al. 1996; Schmidt et al. 2004). If the compound is biodegraded, it is converted to ^13^CO_2_ which leads to changes of the ^13^C/^12^C carbon isotope ratios of the total CO_2_ and the carbonate system. Such a change of the stable isotope ratio therefore indicates the mineralization of the substrate, which can be used to calculate biodegradation rates. However, only a few ^13^C-labeled substrates such as glucose and acetate are commercially available, but they cannot represent the complexity of DOC. Complex substrates, however, cannot be labelled which has hindered the wide use of stable isotope technologies in determining microbial degradation rates in BAC filters, so far.

Dong et al. (2017) presented a simple and cost-effective method for evaluating microbial mineralization of any kind of organic carbon to CO_2_ by reverse stable isotope labelling (RIL). The method is based on adding ^13^C-labelled bicarbonate buffer to the incubation medium at a molar ratio of approximately ten atom percent. Since the degradation of non-labelled organic carbon leads to the evolution of ^12^CO_2_, the ^13^C-stable isotope ratio of the total inorganic carbon (TIC) in the system drops accordingly. Microbial mineralization rates can thus be derived following mass-balance calculations (Dong et al. 2017; Schulte et al. 2019). The advantage of the reverse stable isotope labelling lies in the high sensitivity and precision of ^13^CO_2_/^12^CO_2_ measurements with stable isotope ratio analysis allowing for the detection of very small amounts of CO_2_ evolution. Furthermore, one can use this sensitive technology without the need of applying stable isotope-labelled organic compounds, which are often extremely expensive.

Here, we evaluate the use of reverse stable isotope labelling (RIL) to assess the degradation of adsorbed organic carbon in activated carbon filters for drinking water production. The biodegradable fraction of the organic carbon was measured as released CO_2_ (Korotta-Gamage and Sathasivan 2017a, b). As examples for the applicability of RIL in monitoring biodegradation in BAC filters, we demonstrate the effects of typical water operations, such as backwashing and different BAC filter materials, on microbial activities and removal of organic carbon.

## Materials and methods

### Setup of BAC filters

We used three parallel BAC filters (BAC filters 1–3) belonging to a test facility (RWW, Rheinisch-Westfälische Wasserwerksgesellschaft mbH, Mülheim, Germany) treating raw water from River Ruhr for drinking water production. In the water production facility, the river water is firstly filtered by slow sand filtration where labile organic carbon is removed, followed by ozonation where the DOC is oxidized and becomes more bioavailable. After ozonation, the water runs through three parallel multilayer filters that remove particulate impurities. Each multilayer filter is connected to one BAC filter, respectively. BAC filters 1 and 2 were filled with fresh, heterogeneously sized granular activated carbon (GAC) NORIT^®^ GAC 830 (Cabot Corporation, USA) and filter 3 with fresh, evenly sized extruded activated carbon (EAC) NORIT^®^ ROW 0.8 Supra (Cabot Corporation, USA).

The three BAC filters had identical reactor volumes (20 cm diameter, 200 cm height) and each of them was divided into two phases, the upper surface water part and the lower BAC filter bed. They had different activated carbon bed depths, i.e. filter 1: 40 cm upper water + 160 cm GAC particles; filter 2: 25 cm upper water + 175 cm GAC particles; and filter 3: 55 cm upper water + 145 cm EAC particles. The filters were percolated with the pretreated water from top to bottom. Correspondingly, the empty bed contact times (EBCT) were ~ 10 min, ~ 18 min, and ~ 15 min, respectively (if not specified). The pH of the inflow water was around 7.15 and the temperature 18.6 °C.

### RIL experiments

Activated carbon was sampled from the surface of the three filter beds at several intervals. Since the BAC filters were operated indoors, the inflow water temperatures did not significantly change during the whole experiments. At the end of the experiment, activated carbon was sampled at various depths of the filter beds (0 cm, 20 cm, 40 cm, 80 cm, and 140 cm, measured from the top of the activated carbon bed) in order to determine the vertical distribution of microbial activity. The activated carbon particles (3 g wet weight each) were transferred to three replicate 250 mL serum bottles filled with 50 mL influent water. A large air headspace (200 mL) ensured oxic conditions in the bottles. For each microcosm, NaH^13^CO_3_ and NaH^12^CO_3_ (Sigma-Aldrich Co.) at a molar ratio of 10:90 were injected separately through the stopper to produce a final concentration of 10 mM bicarbonate in the medium. Addition of such amounts of bicarbonate did not change the initial pH values of the water. The organic carbon in each microcosm mostly originated from the DOC adsorbed to the activated carbon particles during the prior operation and to a smaller extent from the DOC dissolved in the used water. All bottles were incubated for at least 24 h at 17 °C in the dark to mimic the temperature conditions of the AC filters.

### Isotope Ratio Mid-Infrared Spectroscopy (IRIS)

Carbon isotope ratios of CO_2_ were measured as described previously (Dong et al. 2017). Briefly, liquid samples (0.5 mL) were taken with a syringe through the stopper of the incubation bottles and injected into closed 12 mL Labco Exetainer vials (Labco Limited, Lampeter, Wales, UK.). The vials were pre-filled with 85% phosphoric acid (50 µL) and flushed with CO_2_-free synthetic air (CO_2_ < 0.1 ppm, Air Liquide, Oberhausen, Germany). Then, the liberated CO_2_ gas in the headspace of these samples was analyzed using a Thermo Fisher Delta Ray CO_2_ Isotope Ratio Infrared Spectrometer (IRIS) with Universal Reference Interface (URI) Connect (Dong et al. 2017; Fischer and Lopez 2016). IRIS is capable of measuring both carbon and oxygen isotope compositions of CO_2_ by comparing various CO_2_ isotopologues of the sample in air (i.e. ^12^C^16^O^16^O, ^13^C^16^O^16^O, and ^12^C^16^O^18^O) (van Geldern et al. 2014). Before measurements, the IRIS was calibrated using two CO_2_ reference gases for carbon isotope ratios: one with *δ*^13^C values of − 9.7‰ [*x*(^13^C) = 1.10%, Thermo Fisher, Bremen, Germany] and the other with *x*(^13^C) = 10% (Sigma-Aldrich, Taufkirchen, Germany) which was also used as working reference gas. The *δ*^13^C values were reported as average of 5 min measurements.

### Calculations of microbial activities

By artificially elevating the background isotopic abundances of ^13^CO_2_, a release of ^12^CO_2_ can be detected as a change of the ^12^C/^13^C-isotope ratio. Mass-balance equations allow then to calculate the amount of released CO_2_ over time.

All carbon isotope ratios (^13^C/^12^C, referred to as *R*) obtained from IRIS were reported in conventional delta notation (*δ*^13^C) as per mil (‰) values, relative to the Vienna Pee Dee Belemnite (VPDB) standard (Eq. 1).

1 $$\delta {}^{13}C = \frac{{R_{sample} }}{{R_{VPDB} }} - 1$$

where 0.0111802 is the ratio of ^13^C/^12^C in the VPDB standard. To facilitate the comparison of results which are obtained from samples highly enriched in ^13^C, all carbon isotope ratios were converted into ^13^C atom fractions (*x*(^13^C)) [%] from *δ*^13^C values (Coplen 2011).

CO_2_ production was calculated from changes in carbon isotope ratios based on isotope mass balance consideration (Eq. 2) (Dong et al. 2017; Schulte et al. 2019).

2 $$CO_{2produced} = \frac{{\left( {CO_{{2{\text{bicarbonate solution}}}} + CO_{2natural} + CO_{2air} } \right) \times \left( {x\left( {{}^{13}C} \right)_{background} - x\left( {{}^{13}C} \right)_{total} } \right)}}{{x\left( {{}^{13}C} \right)_{total} - x\left( {{}^{13}C} \right)_{produced} }}$$

where $CO_{2background }$ is the initial amount of total inorganic carbon in the system [mmol], $CO_{2produced }$ is the CO_2_ released from microbial mineralization of DOC [mmol], $x({}^{13}C)_{total}$ is the final carbon isotope ratio of the total inorganic carbon in the system, $x\left( {{}^{13}C} \right)_{background}$ is the initial carbon isotope ratio of total inorganic carbon in the system, $x\left( {{}^{13}C} \right)_{produced}$ is the carbon isotope ratio of CO_2_ released by microbial production [assumed to be 1.1% based on the abundance of ^13^C in nature (Aelion et al. 2009)], $CO_{{2{\text{bicarbonate solution}}}}$ is the amount of inorganic carbon in the added bicarbonate solution [mmol], $CO_{2natural}$ is the amount of inorganic carbon in the inoculum [mmol], $CO_{2air}$ is the amount of inorganic carbon in the headspace [mmol].

A comparison of different fitting models revealed that a second-degree polynomial matched the CO_2_ production curves (i.e. mineralized DOC) based on measurements at more than five time intervals in the first 24 h of incubation. The slopes of the regression curves were further normalized per liter of volume of BAC filters and provided as the microbial degradation rates.

### Analytical methods

DOC concentrations in water samples were determined using a total organic carbon analyzer (Shimadzu TOC-V CPN) equipped with an auto sampler (Model ASI-V, Shimadzu). For a rough estimation of the total cell counts on the activated carbon samples, detachment of bacteria was performed with a Tween-80 solution (Fluka, 0.1% final concentration) via 5 min ultrasonication and 3 min vortex. The detached bacteria were then filtered through polycarbonate filters with a pore size of 0.22 μm followed by DAPI (4′,6-diamidino-2-phenylindole) staining and visual enumeration with fluorescence microscopy (Lunau et al. 2005).

## Results

### Effect of AC types on microbial activities

**Fig. 1:**
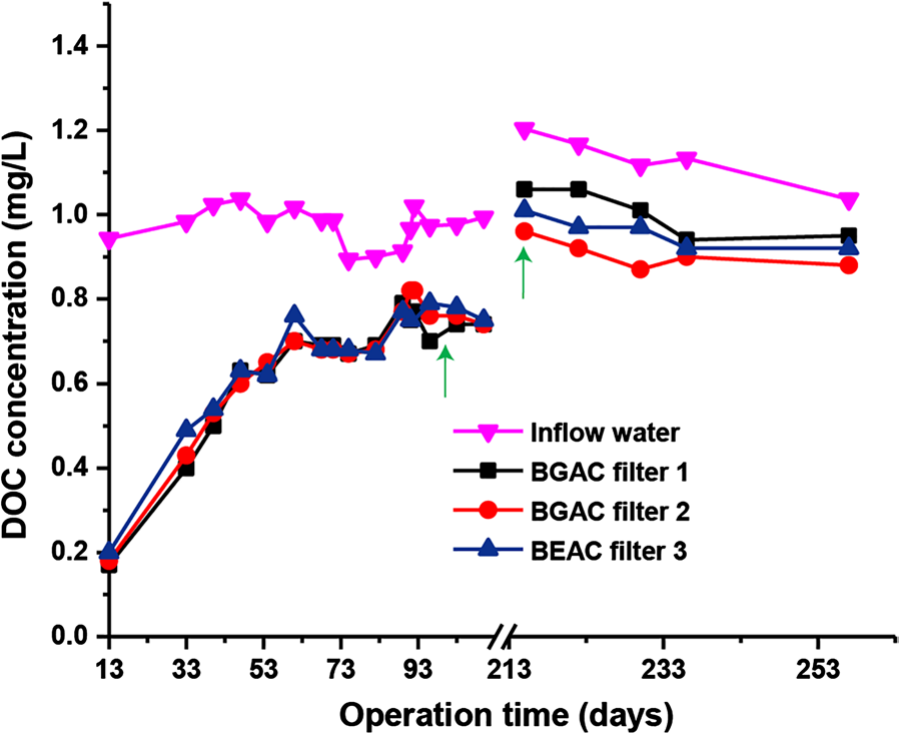
Influent and effluent DOC concentrations of the three BAC filters during two-time periods: I. summer period since May 2016 for 110 days (Day 0–Day 110) and II. winter period since December 2016 for 42 days (Day 215–Day 255). Arrows indicate the backwashing at Day 96 and changing of flow rates at Day 215, respectively

During the summer period from May to August 2016 (110 days), the DOC concentrations in the influent waters of the three BAC filters ranged from 0.89 to 1.03 mg/L (Fig. 1). DOC concentrations were higher during the winter period from December 2016 to January 2017 (42 days) ranging from 1.03 to 1.20 mg/L. The increased DOC in autumn and winter is a common phenomenon caused by e.g. decay of fallen leaves.

The removal of DOC by the BAC filters developed in three stages (Korotta-Gamage and Sathasivan 2017a; Simpson 2008). At the start of the experiment, a significant reduction of around 0.8 mg/L DOC between influent and effluent was observed for all filters (Fig. 1). This first phase (days 0–33) can be interpreted as a strong physical adsorption. As the filter bed size of filter 3 (145 cm) was 10 and 20% smaller compared to filters 1 and 2 (160 and 175 cm), the DOC adsorption rate of filter 3 per volume of extruded activated carbon was much higher than of granular activated carbon in filters 1 and 2.

In the second phase (days 33–61), removal of DOC leveled off which is explained by a gradual saturation of adsorption sites and concurrent establishing of biological degradation. After around 61 days, the third steady state phase started, when around ~ 25% DOC was removed by all three filters regardless of the types of activated carbon. The DOC reduction at this steady stage is mainly attributed to microbial degradation but also to stable adsorption, which, however, are difficult to distinguish (Gibert et al. 2013; Korotta-Gamage and Sathasivan 2017b).

**Fig. 2:**
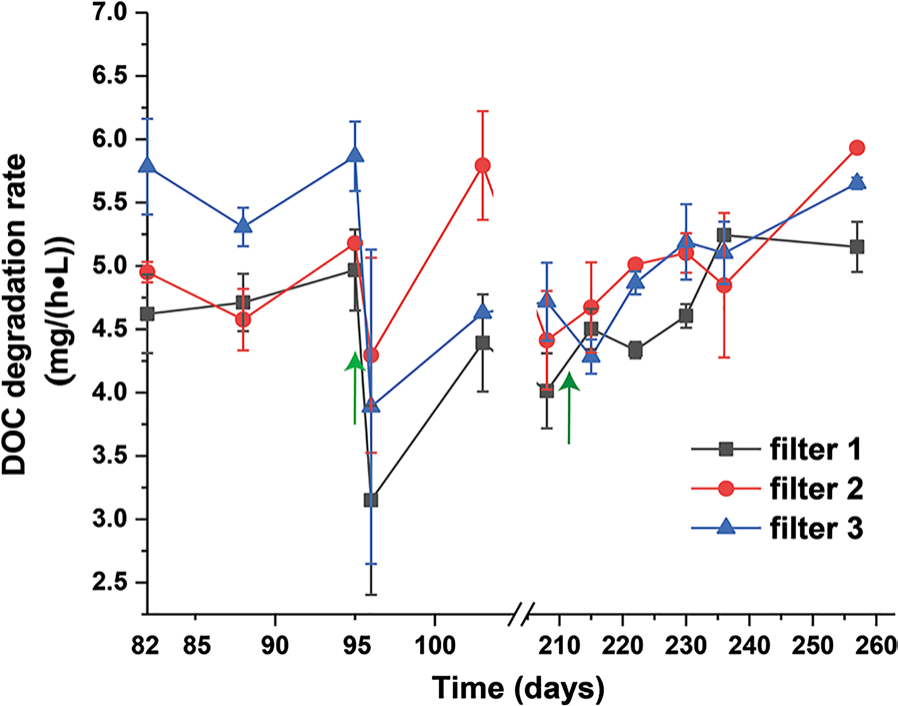
Effect of backwashing and flow rate on microbial degradation rates in BAC filters. Data depict means and standard deviations of three parallel incubations for RIL. Arrows indicate the backwashing at Day 96 and changing of flow rates at Day 208. Filters 1 and 2 are filled with granular activated carbon whereas filter 3 is filled with extruded activated carbon

During the third phase (days 82–95), activated carbon samples were taken from the top surface of the filter beds to determine both biomass densities and DOC degradation activities represented as CO_2_ production rates. At the first two sampling days, the degradation activities per liter in filters 1 and 2 (filled with granular activated carbon) were equal at around 4.8 mg/h, while the material taken from filter 3 (filled with extruded activated carbon) showed ~ 1.2 times higher degradation rates of 5.8 mg/h (Fig. 2). This disagrees with similar bacterial densities in the three filters (Table 1) and might be related to the higher adsorption kinetic and specific capacity of the extruded activated carbon.

**Table 1:** Total cell count per liter volume of the three BAC filters

Day	Cell number (10^11^/L)		
	Filter 1	Filter 2	Filter 3
82	7.7 ± 1.8	7.3 ± 2.6	7.4 ± 1.2
88	8.1 ± 2.7	7.3 ± 1.0	7.4 ± 0.8
95	8.6 ± 1.4	7.3 ± 1.0	8.2 ± 1.6
96	4.5 ± 0.9	4.7 ± 1.0	4.3 ± 0.8
103	8.6 ± 2.3	7.8 ± 2.6	7.0 ± 1.6

The biomass densities were measured from activated carbon samples taken from the top surface of the filter beds. Data show means of three replicates ± standard deviation. Filters 1 and 2 were filled with granular activated carbon whereas filter 3 was filled with extruded activated carbon

### Spatial distributions of microbial activities

**Fig. 3:**
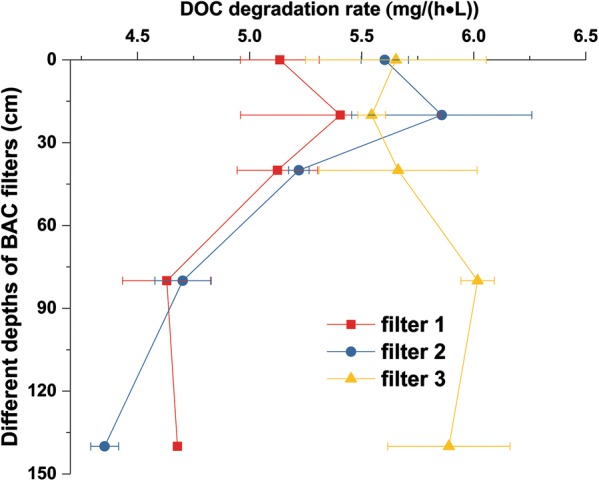
Microbial DOC degradation rates as a function of depth in the BAC filters. Depth 0 corresponds to the top of the filter bed. Data depict means and standard deviations of three parallel incubations. Filters 1 and 2 are filled with granular activated carbon whereas filter 3 is filled with extruded activated carbon

At the end of the experiment (Day 258), we sacrificed the reactors and sampled activated carbon at different depths to characterize the spatial distribution of microbial activities along the filter beds. In BAC filters 1 and 2, the highest microbial activities were found at the second sampling point located 20 cm below the top surface layer (~ 5.4 and 5.9 mg CO_2_–C/(h L), respectively). They decreased by 14% and 26% to the bottom of filters 1 and 2, respectively (Fig. 3).

### Effect of flow rates on microbial activities

In order to test if transport of DOC to the microorganisms on the BAC particles limits the biodegradation activity, we changed the flow rates of the three filters (Fig. 1). At day 208 (data not shown), the flow rate of filter 1 was increased from 300 to 450 L/h, the flow rate of filter 2 was decreased to 150 L/h, while filter 3 remained unchanged at 300 L/h. The DOC removal by the three filters showed clear responses to changing flow rates, with corresponding removal efficiencies of filter 3 remaining at around 16%, filter 1 decreasing to 12% and filter 2 increasing to 20% after 1 week (at day 215) (Fig. 1), respectively. This systematic change indicates that longer contact times of the water (lower flow rate) to the BAC increased the removal of organic matter.

### Effect of backwashing on microbial activities

At Day 96, our filters were backflushed twice (5–6 min each) using clean drinking water at flow velocities of 2.8–3.2 m/h. After backwashing, cell numbers of the filters 1, 2, and 3, decreased by 45%, 33%, and 43%, respectively (Table 1). Similarly, the CO_2_ production rates declined by 36%, 17%, and 33%, respectively (Fig. 2). Only 1 week later, both parameters fully recovered to values before backwashing.

## Discussion

So far, there are only indirect methods available to measure microbial degradation activities of organic carbon adsorbed to solid matrices. Here, we demonstrated that reverse isotope labelling (RIL) can be applied to directly monitor microbial degradation activities and DOC removal efficiencies for biological activated carbon (BAC) filters of a drinking water treatment plant. To this end, we applied reverse stable isotope labelling (RIL) for assessing minute rates of microbial CO_2_ release (Dong et al. 2017). With the aid of mid-infrared laser spectroscopy, we comprehensively demonstrated effects of AC types, spatial distributions, flow rates, and backwashing on microbial activities in BAC filters.

### Effects of AC types

The data (Fig. 1 and Table 1) indicate that the microorganisms in filter 3 had much more adsorbed organic substrate available per liter volume of AC, most likely originating from the better adsorption process of the extruded AC. This observation suggests that the DOC removal efficiencies were not only affected by the adsorption/desorption equilibrium of DOC to the respective AC material (Korotta-Gamage and Sathasivan 2017b; Yapsakli and Cecen 2010), but also by the correlated support of the microorganisms with organic substrate for biodegradation.

### Spatial distributions of microbial activities

The distribution of the DOC mineralization potential (Fig. 3) may be related to a stratification of different sizes of the activated carbon particles. This is because microorganisms are mostly located on the fine particles fraction (Urfer and Huck 2001). Similar observations were made for many column experiments and were attributed to the degradation of the organic substrate and growth of the microorganisms towards the inlet (Gibert et al. 2013; Velten et al. 2011). Indeed, the regular backwashing of the two filters 1 and 2 resulted in an accumulation of smaller particles in the upper parts (Frank et al. 2015). Hence, it seems that the fine particles of NORIT^®^ GAC 830 lead to a higher DOC removal either due to the smaller physical size and better solute transport properties to the attached biomass or due to a higher accumulation of biomass on smaller particles (Velten et al. 2007). For filter 3, filled with the extruded activated carbon, we observed an almost constant microbial activity along the filter depth. Since the extruded activated carbon had a very homogeneous size distribution of the particles with a round and compact shape and no fines, this type of activated carbon did not undergo a physical size separation during backwashing. We therefore attribute the gradient of microbial activity in filters 1 and 2 to the size distribution of the activated carbon particles rather than to an effect of enhanced growth at the filter inlet. Thus, the material of filter 3 showed superior properties for a more homogenous distribution of microbial activity and filter performance. The spatial distributions of microbial activities may be also related to oxygen gradients along the filters. This is because microbial DOC degradation would consume up oxygen at the near inflow site. Microbes that live far from the inflow site would be limited by oxygen availability, leading to the decrease in microbial activities.

### Effect of flow rates

Changing the flow rates led to only small differences in the microbial degradation activity measured with RIL (Fig. 2) indicating that the microbial degradation activities in our experiments are limited by the desorption rates of organic carbon from the BAC particles or the microbial biomass that established on the AC. This aligns with other studies indicating that the biodegradation reaction time on the filters could be an important limiting factor for DOC removal (Pramanik et al. 2015).

### Effect of backwashing

Backwashing is important to avoid clogging of AC filters during long-term operation in drinking water production (Kim et al. 2014). However, it is not known if the removal of biomass during backwashing impairs the microbial degradation properties of the bio filters. In our experiments, the performances of microbial activities recovered in 1 week (Fig. 2). This suggested that the backwashing reduced the DOC degradation activity because microbes were washed out (Frank et al. 2015; Kim et al. 2014). Nevertheless, the filter functions were mostly maintained and recovered quickly.

Overall, we showed that degradation of organic carbon adsorbed to solid particles could be well assessed by measuring CO_2_ development or mineralization rates with RIL. This is especially interesting for quantifying DOC degradation on activated carbon, which is a strong adsorbent prohibiting the determination of substrate concentrations. Since adsorption does not lead to CO_2_ production, the RIL method allowed distinguishing adsorption and biodegradation in complex environmental processes. This can be used to analyse microbial activities from environmental samples or to improve technical processes such as activated carbon filtration by e.g. identifying if biodegradation processes are limiting DOC removal efficiencies. The easy analysis of the RIL with bench top analytical instruments provides a reliable and comparable way to quantify degradation processes on solid matrices such as activated carbon. Compared to other methods for assessing degradation activities that deliver only relative units, RIL provides degradation rates in mg CO_2_/(L h), which makes such data comparable between different laboratories and allows quantifying and calculating mass balances.

## Data Availability

The authors declare that all data supporting the findings of this study are available within the article and its supplementary information files, or from the corresponding authors upon request.
